# Mapping of Urinary Schistosomiasis in Anambra State, Nigeria

**DOI:** 10.5334/aogh.2393

**Published:** 2019-04-02

**Authors:** Yvonne E. Ndukwe, Rose N. N. Obiezue, Ifeanyi Oscar N. Aguzie, Joy T. Anunobi, Fabian C. Okafor

**Affiliations:** 1 Parasitology and Public Health Unit, Department of Zoology and Environmental Biology, University of Nigeria, Nsukka, Enugu State, NG; 2 Science Laboratory Technology Department, Federal Polytechnic, Idah, Kogi State, NG

## Abstract

**Background:**

Mapping the distribution of parasitic diseases in time and space has a pivotal role to play in their control.

**Objectives:**

This study mapped urinary schistosomiasis in Anambra State.

**Methods:**

Sampling covered the three senatorial districts, Anambra North, Anambra Central and Anambra South. However, only nine of the 21 local government areas (LGAs) and one town in each LGA were covered. A geographic information system (GIS) was used to map the distribution of schistosomiasis in the state. With the aid of GIS, the distance of the towns to water bodies was calculated. A total of 450 urine samples collected from the nine LGAs were examined for haematuria and Schistosoma haematobium eggs. A questionnaire was used to assess exposure and risks status to infection. The urine samples were examined for haematuria using dipstick and microscopy.

**Findings:**

Overall prevalence of infection in the study was 2.9% and 5.5% for microscopy and haematuria, respectively. Prevalence of schistosomiasis was different between the districts, and this was statistically significant (χ^2^ = 7.763, p = 0.021). Prevalence of urinary schistosomiasis in the towns had a significant negative linear relationship with distance to water body (r = –0.767, p = 0.016). Based on infection status from microscopy, the adjusted odds of infection in fishers was over 103 times higher than in students; the difference was significant statistically (AOR = 103.0443, 95% CI = 4.6278–7093.972, p = 0.0114). People who washed things in stream had 12 times significantly greater odds of infection than those that did not (AOR = 12.4585, 95% CI = 1.9590–258.8108, p = 0.02542). The distance of respondents to stream was a major determinant of infection with urinary schistosomiasis in the state. Those who lived close to water were approximately 1131% more likely to be infected than those who lived far from water bodies (AOR = 11.3157, 95% CI 2.2473–90.6889, p = 0.00713).

**Conclusions:**

Anambra State is endemic for urinary schistosomiasis. There is therefore a need for focal studies; and there may probably be a need to design a health program aimed at controlling the infection in focal areas in the state. The study also provides relevant information for designing a plan of action for the selective integrated and targeted control of urinary schistosomiasis in the LGAs.

## Publisher's Note

A correction article relating to this publication can be found here: http://doi.org/10.5334/aogh.2540

## Introduction

Improved understanding of subnational geographic variations in health status and access to resources within countries is increasingly recognized as central to meeting development goals [1]. In pursuance of this goal, many studies explore the potential of geostatistical approaches for the production of interpolated surfaces derived from global positioning system (GPS) cluster located survey data and use it to produce gridded patterns of disease status and risk factors. These interpolated surfaces aid in mapping of many endemic diseases like malaria, soil-transmitted helminth infections and schistosomiasis.

Schistosomiasis is one of the major neglected tropical disease (NTD). The disease is closely related with conditions of poverty, poor sanitation and unavailability of clean water. An estimated 240 million people in 78 countries are infected [2]; and approximately 85% of world cases occur in Africa [3]. It remains a major public health problem in poor communities with enormous consequences for development. Control of the disease continues to gain momentum in Nigeria with increasing commitment from the federal government and a number of foreign and national nongovernmental organisations (NGOs) to provide the funding, and from pharmaceutical companies that provide drugs. Experts say that beyond these helps, the knowledge of the distribution of disease is essential for developing an efficient and adequate implementation strategies for control and to design drug packages for distribution in endemic foci. An accurate estimate of the proportion of a population affected by a disease is important for prioritizing the control of that disease relative to another and to allocating resources in control and prevention of the disease [4].

Schistosomiasis is one of the most widely spread among the parasitic helminthic infections that affect man. It is an occupational risk encountered in rural areas of developing countries, where portable water is scarce. The disease is indicated by the presence of blood in urine and sometimes by pains on urinating or after urinating. Man contacts the disease when he comes in contact with infected water bodies while carrying out necessary daily activities such as farming, fishing, laundry, bathing and swimming [5]. These socioeconomic activities and symptoms are not uncommon among the inhabitants of Anambra State, Nigeria. With many rivers, ponds, irrigated farming and burrow pits, Anambra State has diverse freshwater environments that offer numerous favourable habitats for aquatic snails that serve as intermediate hosts.

Mapping the distribution of these diseases in time and space has a pivotal role to play in their control. For this reason, WHO launched the NTDs atlas initiative and is currently jointly implemented with the Food and Agriculture Organization of the United Nations (FAO) in the framework of the programme against NTDs. Schistosomiasis is one of the NTDs, so this work is designed to be a major contribution toward the initiative in Anambra State. Brooker et al. [6] said that mapping of NTDs, including schistosomiasis, is the way forward in the control of these NTDs because in the past, national reporting on NTDs has been incomplete and unreliable because of weak disease surveillance system, often necessitating dedicated surveys to be undertaken. In the case of schistosomiasis, one of the first steps of the Schistosomiasis control initiative (SCI) in designing country programmes was to conduct national prevalence surveys to identify communities requiring mass treatment with praziquantel. Outside SCI supported countries, national surveys of schistosomiasis and soil-transmitted helminth (STH) are often scarce. Despite these efforts, there remains a considerable mapping requirement to support this global NTD control initiative because it is believed that as control becomes successful in reducing transmission, there will be an increasing requirement to conduct mapping surveys to access the nature of mass drug administration (MDA) required, either to continue the present treatment schedule or to lessen it. This work is designed to be a major contribution toward the SCI in Anambra State because the area has many rivers, ponds, irrigated farming and burrow pits; Anambra State has diverse freshwater environments that offer numerous favourable habitats for aquatic snails that serve as intermediate hosts to this parasite. This study will be instrumental in the elimination of this disease in Anambra State and Nigeria at large. The specific objectives include to (i) assess the current distribution of urinary schistosomiasis in Anambra State, Nigeria; (ii) use prevalence studies to stratify the study area according to risks; and (iii) identify associated risk factors of the infection in Anambra State.

## Materials and Methods

### Study Area

**Figure 1:**
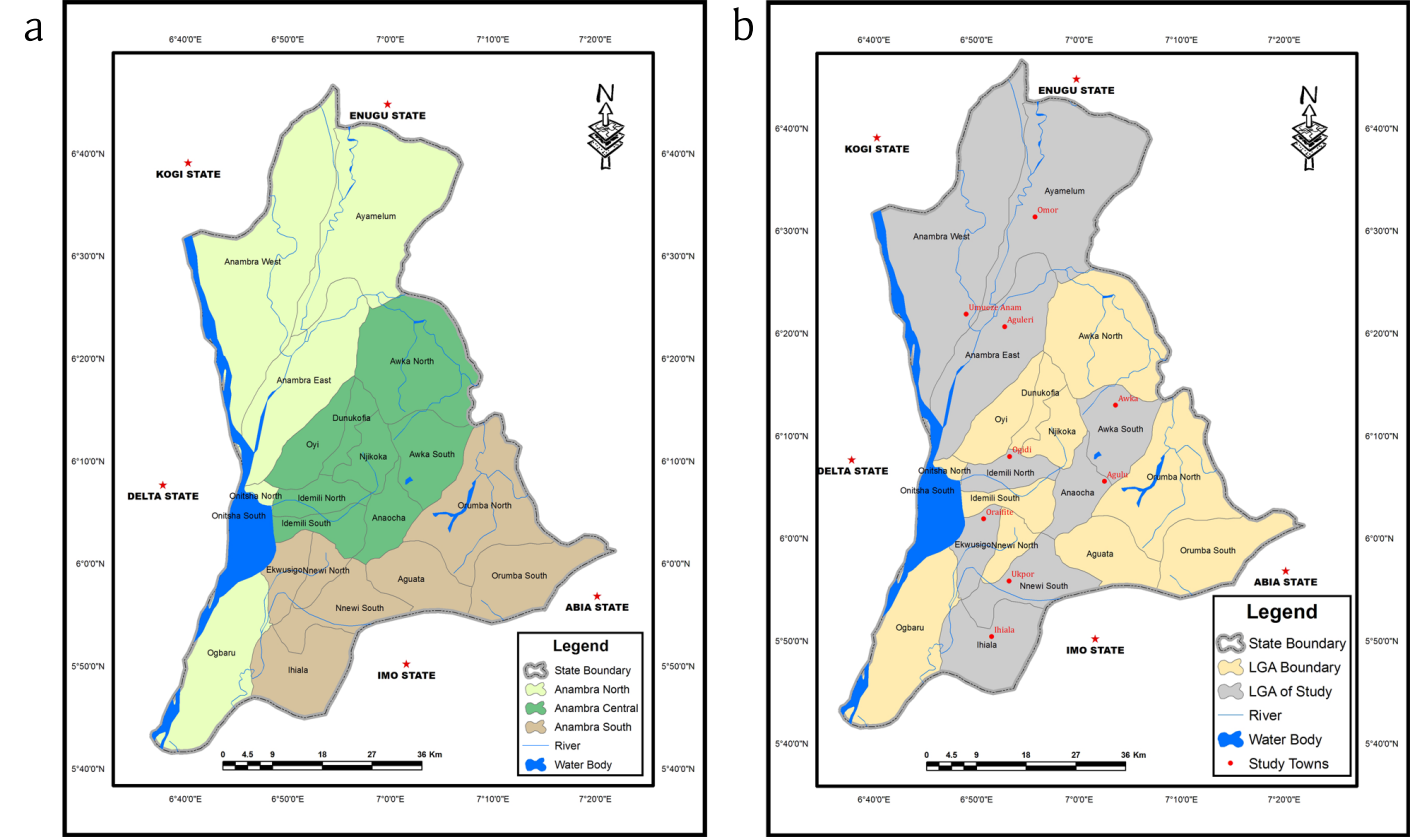
Map of Anambra state showing study areas in **(a)** senatorial districts, **(b)** local government areas/towns.

Anambra State is in southeastern Nigeria and lies between latitude 5° 40′ 00″ N and 6° 50′ 00″ N and longitude 6° 40′ 00″ E and 7° 20′ 00″ E. It is bounded by Delta State to the west, Imo State and Rivers State to the south, Enugu State to the east and Kogi State to the north. It has three senatorial districts, namely Anambra North, Anambra Central and Anambra South. The senatorial districts were used as yardstick for partitioning of the study area. Three local government areas (LGAs) were randomly chosen from each of the senatorial districts. In each of these LGAs, a town was randomly chosen (Figures 1a, b). Thus, the study was conducted in nine randomly selected communities from nine LGAs. The towns were Agulu (6.0923°N, 7.0411°E), Ogidi (6.1328°N, 6.8871°E), Awka (6.2161°N, 7.0590°E), Aguleri (6.3440°N, 6.8787°E), Umueze-Anam (6.3642°N, 6.8163°E), Omor (6.5224°N, 6.9281°E), Ukpor (5.9305°N, 6.8859°E), Ihiala (5.8399°N, 6.8578°E) and Oraifite (6.0314°N, 6.8447°E). The area has typical semitropical rainforest vegetation, characterized by fresh water swamps. It has a humid climate with a temperature of about 30.6°C (87°F) and a rainfall between 152 and 203 centimeters annually. The major rivers in the state are River Niger, Omambala, Ulasi and Ezu River. There are other smaller streams, lakes, ponds and burrow pits. With many rivers, ponds, irrigated farming and burrow pits, Anambra State has diverse freshwater environments that offer numerous favourable habitats for aquatic snails that serve as intermediate hosts to *Schistosoma* [7].

### Study Population

The population of Anambra State according to a 2006 population census was 4,055,048 [8]. The sample size was calculated using the Sloven’s formula {\rm{s}}= {\textstyle{N \over {1 + N{{(e)}^2}}}}. where N = population size, e = error limit. At an error rate of 0.05, a sample size estimate of 400 was considered acceptable [9]. However, a total of 450 persons were recruited for the study.

### Ethical Clearance

Permission was obtained from Anambra State’s Ministry of Health to carry out the work in the state. The ethical clearance number assigned to the study was MH/COMM/552/28. Before the commencement of the study, the objectives and plan were explained to the heads of the selected communities in order to get their cooperation and permission to conduct the survey. Individuals who voluntarily agreed to participate were included in the study.

### Research Design

The study made an attempt to provide a preliminary map of schistosmiasis distribution in Anambra State. This study followed a cross-sectional, community-based design. The three senatorial districts of Anambra State were covered. A “one town per LGA” preliminary sampling approach was employed, where one town per LGA from three LGAs were randomly selected from each senatorial district. The point of entry of the researchers into any town served as start points of sample collection. Attempts were made to cover an area where there were temporary dense human population, mainly schools and markets. Prevalence of urinary schistosomiasis and the exposure and risks status in the communities and populations was classified. A global positioning system (GPS) was used to get the accurate placement of the communities via their longitudes and latitudes. Aided by a geographic information system (GIS), a urinary schistosomiasis map was generated that showed the areas with infections. The GIS also showed the relationship between water bodies and communities with infection. *Stream* was generally used in reference to standing water such as river, pond and stream. The study lasted from January to June 2017.

### Data Collection

The nine communities selected in the study area were visited on a pilot study. There, some elderly people were approached for discussions on relevant issues about their communities. Questions were asked about their sources of water for drinking and other domestic activities. Information on nearness to existing streams and rivers were obtained. The occupation of the people was also considered. A total of 400 questionnaires were given to the individuals who voluntarily consented. The questionnaires contained questions relevant to the study such as participant’s age, sex and occupation, contact with standing water and other water contact activities. The questions were close ended and written in English language. Where a respondent had problems with reading or understanding English, questions were either read out or interpreted to them, and responses were recorded as the case may be. Parents and guardians responded for children or wards who were too young to respond (mainly those within 0–10 years).

Screw-capped, prenumbered, tagged and sterilized plastic containers were given to each person, to collect urine samples. The subjects were instructed on how to collect the urine samples. Urine samples were collected between the hours of 9:00 a.m. and 2:00 p.m. They were instructed to collect midstream urine not less than a volume of 10 mL and have the last few drops of the urine passed included in the bottles. The last drops often contain the highest number of eggs [10]. Females in their monthly periods were marked and excluded from visible haematuria counts. This is necessary in order to avoid false positive results [11]. Urine samples were transported within five hours of collection in suitable cool boxes at temperature between 4°C and 6°C for subsequent examination at the Parasitology Laboratory, Department of Zoology and Environmental Biology, University of Nigeria, Nsukka, Enugu State, Nigeria.

### Parasitological Examination

Urine samples were examined for haematuria using dipstick (Medi-Test Combi 9, Macherey-Nagel GmbH & Co. KG). Reading was taken by two observers who were strictly guided in order to achieve independent reports. Thereafter, the urine sample was centrifuged at 3000 rpm for three minutes, and the sediments were examined under the microscope for the presence of *S. haematobium* eggs as described by Cheesbrough [10]. Negative or positive reports from microscopy were validated by an independent observer. *Schistosoma haematobium* parasite was identified based on the morphology of the egg, which has a diagnostic terminal spine (see Supplementary File 1). Microscopic examination was done at × 10 and × 40 objective lens.

### Statistical Analysis

Schistosomiasis prevalence was compared using chi-square analysis. Mixed-effect logistic regression model was used to evaluate the odds of infection with *Schistosoma haematobium* in relation to demographic and water contact activities. Univariate logistic regression was used to estimate the crude odd ratios of infection associated with twelve variables (see Supplementary File 2). Purposeful selection and backward selection procedures were used to assist manual selection approach to choose appropriate variables for evaluation of the adjusted odds of *S. haematobium* infection [12,13]. Four out of the 12 initial independent categorical variables were eventually chosen for the final multivariate model for adjusted odd ratio. These were “occupation,” “swimming in stream,” “wash in stream” and “distance to stream.” “Bathe in stream,” which was significant predictors at the initials, was eventually removed due to redundancy in the presence of “wash in stream” and “swim in stream” and its removal improved the model. “Age group” and “occupation” played similar roles in the multivariate logistic regression model, which we understood to mean that the significant crude odds of infection in some age groups from our univariate models may be due to occupation of people in those age groups. Inclusion of “occupation” and “age group” degenerated our model. Therefore “occupation,” which better explained the result when adjusted for other relevant variables, was preferred to “age group.” Statistical significance was considered at 95% probability level (p < 0.05). Data was analysed using R for windows version 3.5.0 and SPSS version 20.0 (IBM Corporation, Armonk, New York, USA).

## Results

### Prevalence of Urinary Schistosomiasis in Anambra State, Nigeria

A total of 450 urine samples were examined for urinary schistosomiasis; 25 (5.5%) and 13 (2.9%) were positive from haematuria and microscopic detection of eggs, respectively. Overall, the prevalence of urinary schistosomiasis was low whether from haematuria or microscopy, but high prevalence of infection was detected when prevalence was considered by the occupation of study participants. Those who had fishing as their occupation were 71.4% positive for haematuria, and 57.1% shed *S. haematobium* eggs from their urine. Compared to the civil servants, traders, students and farmers, this was significantly higher in either cases of haematuria and microscopy (χ^2^ > 50.000, p < 0.001). Prevalence of schistosomiasis was low in the five age groups considered (Table 1). Prevalence of the infection was generally not more than 15.0% in all the age groups, but prevalence of infection from microscopy was significantly different between the age groups (χ^2^ = 11.447, p = 0.022). Prevalence of schstosomiasis was not different between male and female participants.

**Table 1:** Prevalence of urinary schistosomiasis based on haematuria and microscopy.

	Number examined	Number infected (%)	

**Occupation**		Haematuria	Microscopy

Civil servant	28	0 (0.0)	0 (0.0)
Farmer	22	3 (13.6)	2 (9.1)
Student	282	16 (5.7)	6 (2.1)
Trader	28	1 (3.6)	1 (3.6)
Fishing	7	5 (71.4)	4 (57.1)
		**χ^2^ = 50.741, p < 0.001**	**χ^2^ = 63.523, p < 0.001**
**Age (year)**			

0–10	183	7 (3.8)	3 (1.6)
11–20	94	10 (10.6)	4 (4.3)
21–30	45	3 (6.7)	1 (2.2)
31–40	20	3 (15.0)	3 (15.0)
≥41	25	2 (8.0)	2 (8.0)
		χ^2^ = 6.909, p = 0.141	**χ^2^ = 11.447, p = 0.022**
**Sex**			

Male	244	12 (4.9)	7 (2.9)
Female	206	13 (6.3)	6 (2.9)
		χ^2^ = 0.413, p = 0.521	χ^2^ = 0.001, p = 0.978
**Total**	**450**	**25 (5.5)**	**13 (2.9)**

### Geographical Information System (GIS) Map of Urinary Schistosomiasis in Anambra State, Nigeria

**Figure 2:**
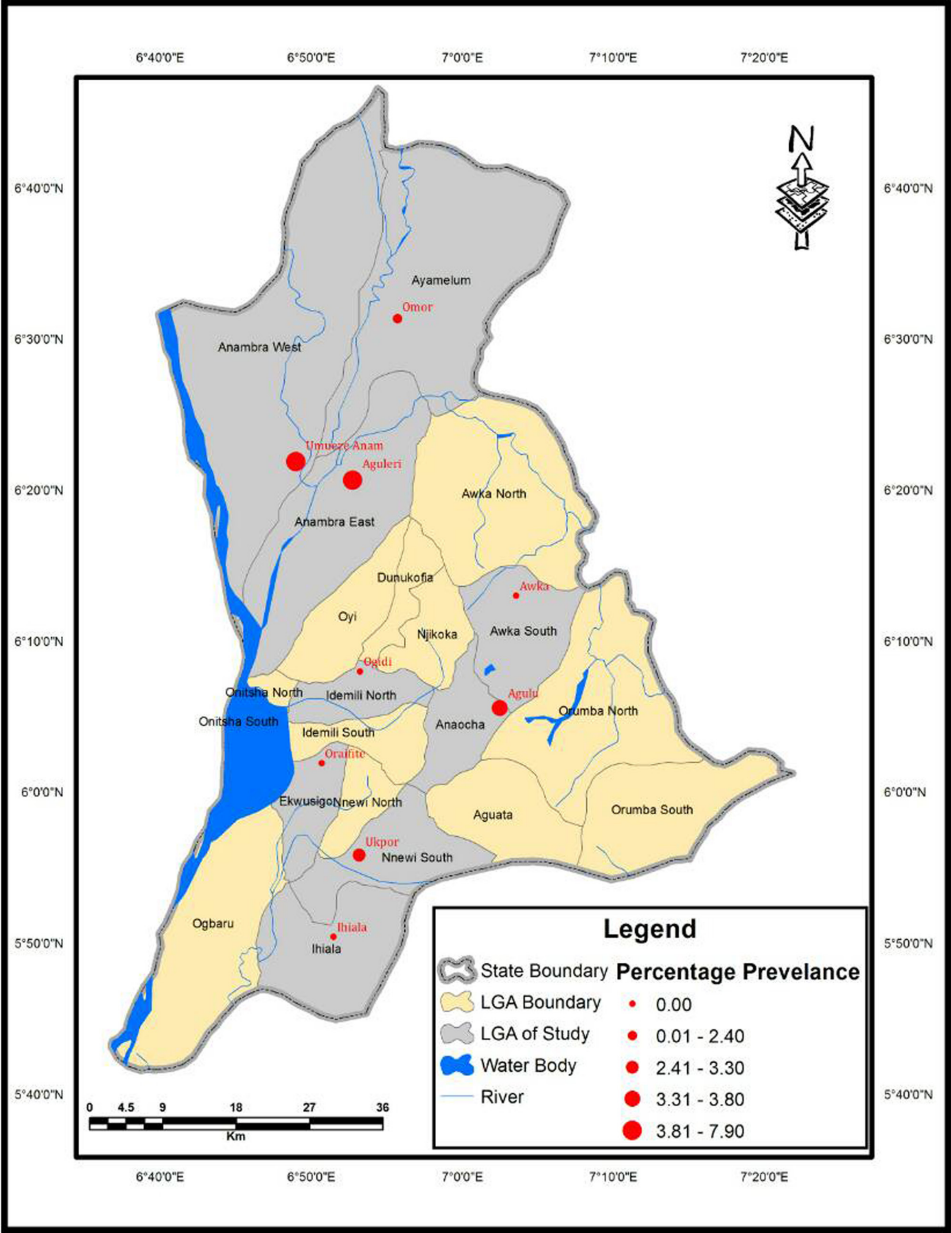
Prevalence map of urinary schistosomiasisbytowns in Anambra State, Nigeria.

**Figure 3:**
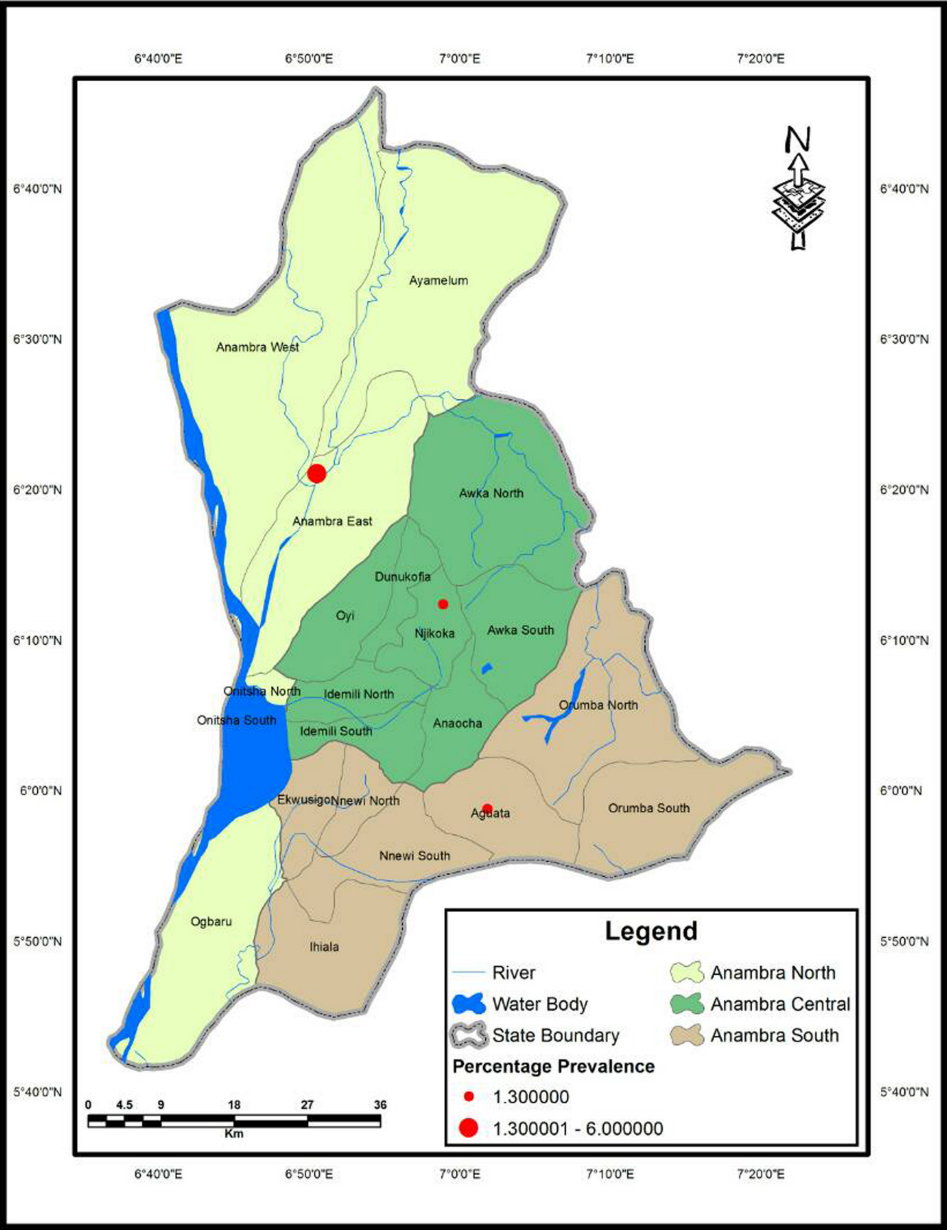
Prevalence of urinary schistosomiasisbysenatorial districts in Anambra State, Nigeria.

Overall, 150 persons were examined per senatorial district. Anambra North had the highest prevalence of urinary schistosomiasis from microscopic detection of eggs in urine, 9 (6.0%); the other two districts each had 1.3% prevalence of the infection (Table 2). The difference in urinary schistosomiasis prevalence was significant between the senatorial districts (χ^2^ = 7.763, p = 0.021). With the aid of GIS, the distance of each town to water bodies was calculated (Table 2). Prevalence of urinary schistosomiasis in each town had a significant negative linear relationship with the distance of the town to a water body; as the distance of the town to a water body reduced, the chances of its inhabitants having *S. haematobium* infection increased (r = –0.767, p = 0.016). The prevalence of urinary schistosomiasis in relation to water bodies is mapped in Figures 2 and 3.

**Table 2:** Distance to water body and prevalence of urinary schistosomiasis in Anambra State.

Locations			Number Infected (%)	Distance to Water (km)

Senatorial District	LGAs	Towns		

Anambra North	Anambra East	Aguleri (*n* = 46)	3 (6.5)	2.409577
(*n* = 150)	Anambra West	Umueze-Anam (*n* = 63)	5 (7.9)	1.2166
	Ayamelu	Omor (*n* = 41)	1 (2.4)	2.142287
			**9 (6.0)**	
Anambra Central	Anaocha	Agulu (*n* = 53)	2 (3.8)	3.258459
(*n* = 150)	Idemili North	Ogidi (*n* = 47)	0 (0.0)	7.522285
	Awka South	Awka (*n* = 50)	0 (0.0)	3.5805
			**2 (1.3)**	
Anambra South	Nnewi South	Ukpor (*n* = 60)	2 (3.3)	2.067764
(*n* = 150)	Ihiala	Ihiala (*n* = 48)	0 (0.0)	8.436227
	Ekwusigo	Oraifite (*n* = 42)	0 (0.0)	6.493389
			**2 (1.3)**	

*n* = number examined. Total number infected and percentage per senatorial district in bold font.

### Questionnaire Responses

Among those who provided urine samples, a total of 367 individuals also responded appropriately to the questionnaires out of the 400 distributed. The remaining 33 were excluded due to nonsubmission of questionnaires and incomplete responses. Table 3 shows the demographic and water contact behaviour of the questionnaire respondents. A larger fraction of the respondents were from Anambra North (45.8%), followed by Anambra Central (34.9%). More females responded (55.9%) than males (44.1%). A majority of respondents (79.0%) used tap as source of drinking water, as opposed to stream (1.9%), rain (7.9%) and sachet water (11.7%). Some of the study participants had contacts with standing water bodies for different purposes. The activities for which they visited water bodies were bathing (20.02%), washing (19.9%), fishing (12.3%), swimming (34.6%) and just visiting (21.0%)

**Table 3:** Overall demographic and water contact activities of questionnaire respondent (*n* = 367).

Variables	Frequency (%)

**Districts**	

Anambra North	168 (45.8)
Anambra Central	128 (34.9)
Anambra South	71 (19.3)
**LGAs/Towns**	

Anambra East/Aguleri	54 (14.7)
Anambra West/Umueze-Anam	61 (16.6)
Ayamelu/Omor	53 (14.4)
Anaocha/Agulu	47 (12.8)
Idemili North/Ogidi	40 (10.9)
Awka South/Awka	41 (11.2)
Nnewi South/Ukpor	26 (7.1)
Ihiala/Ihiala	22 (6.0)
Ekwusigo/Oraifite	23 (6.3)
**Sex**	

Male	162 (44.1)
Female	205 (55.9)
**Age Groups**	

0–10	183 (44.9)
11–20	94 (25.6)
21–30	45 (12.3)
31–40	20 (5.4)
≥ 41	25 (6.8)
**Occupation**	

Civil Servant	28 (7.6)
Farmer	22 (6.0)
Student	282 (76.8)
Trader	28 (7.6)
Fishing	7 (1.9)
**Water Contact**	

*Sources of drinking water*	
Tap water	290 (79.0)
Stream water	7 (1.9)
Rain water	27 (7.9)
Sachet water	43 (11.7)
*Purpose for visiting standing water*	
Bathing	74 (20.2)
Washing	73 (19.9)
Fishing	45 (12.3)
Swimming	127 (34.6)
Visiting	77 (21.0)

*n* = number examined.

### Risks Factors for Urinary Schistosomiasis in Anambra State, Nigeria

Based on infection status from microscopic detection of *S. haematobium* eggs in urine, the adjusted odds of infection in fishers was over 103 times higher than in students; the difference was also significant statistically (AOR = 103.0443, 95%CI = 4.6278–7093.972, p = 0.0114). Farmers and traders were also 13 and 11 times, respectively, more likely to be infected with schistosomiasis than students, though these were not significant at the 0.05 cutoff point (p > 0.05; Table 4). Swimming in streams increased the odds of infection by 21 times, though the difference was not significant statistically (AOR = 0.9147–2562.267, p = 0.1140). People who washed things in stream had 12 times significantly greater odds of infection than those that did not (AOR = 12.4585, 95%CI = 1.9590–258.8108, p = 0.02542). The distance of respondents to stream was a major determinant of infection with urinary schistosomiasis in the State (Table 4). Those who lived close to water were approximately 1131% more likely to be infected than those that lived far from water bodies (AOR = 11.3157, 95% CI 2.2473–90.6889, p = 0.00713). Generally, urinary schistosomiasis infection in the area was associated with presence of water bodies such as stream, pond or river. Other activities not included in the final model but were related to activities in water bodies were risk factors for infection. Activities in freshwater bodies such as bathing in stream, swimming stream, drinking stream water, washing in stream, fishing in stream and distance to stream were each associated with significant crude odds of infection with urinary schistosomiasis in Anambra State (Supplementary File 2). Individually, people in age groups 31–40 years and fishers were at significantly higher crude odds of infection compared to 0–10 years and students, respectively (Supplementary File 2).

**Table 4:** Predisposing factors to infection with urinary schistosomiasis in Anambra State, Nigeria (*n* = 367).

Variable	Status	AOR (95% CI)	P

Occupation	Student	Reference	
	Civil servant	*	0.9949
	Farmer	13.5949 (0.8946–304.7746)	0.0689
	Traders	11.3967 (0.4425–178.3362)	0.0802
	Fishing	103.0443 (4.6278–7093.972)	**0.0114**
Swimming Activity	Swim in stream	21.2710 (0.9147–2562.267)	0.1140
	Does not swim	Reference	
Wash in stream	Yes	12.4585 (1.9590–258.8108)	**0.0254**
	No	Reference	
Distance to stream	Near	11.3157 (2.2473–90.6889)	**0.0071**
	Far	Reference	

OR: Odds ratio. CI: confidence interval. *odd ratio unavailable due to no infection in category, therefore odds not defined.

## Discussion

The study showed that urinary schistosomiasis is endemic in the three senatorial districts of Anambra State. Anambra North had the highest prevalence (6.0%). This may be due partly to the fact that the area is surrounded by many freshwater bodies (including Omambala River and Otuocha River) and several other smaller rivers and streams. Another factor may be because the people are mostly farmers and fishermen, and their lifestyle suggests frequent contact with water through swimming, bathing, fishing, farming and laundry. In the nine communities sampled, four communities–namely Ogidi, Awka, Ihiala and Oraifite–had zero prevalence. This may be as a result of their infrequent contact with water bodies, probably due to the far distance to a water body. The other five communities of Aguleri, Umueze-Anam, Omor, Agulu and Ukpor had the infection, with Umueze-Anam having the highest prevalence (7.9%). This may be because they live very close to water. It may also be as a result of their lifestyle, which include frequent contact with water, poor hygiene, limited access to clean water and subsistence farming [14]. The prevalence map of urinary schistosomiasis in the study area showed that infection was more in Anambra North than in other districts. It also showed that distance to water body was a major determinant of infection. From the map, Umueze-Anam was close to water body (1.2166 km), and it had the highest prevalence (7.9%) followed by Aguleri (2.409577 km), having a prevalence of 6.5%. Places like Ihiala (8.436227 km), Ogidi (7.522285 km) and Oraifite (6.493389 km) that were very far from water bodies had no infection. The study strongly suggests a need to focus attention at the foci of disease transmission. The GIS map obtained provides details about areas where focal studies and control efforts needs to be focused.

The prevalence of urinary schistosomiasis in Anambra State is low. The low prevalence corroborates reports from other studies in the area. Ugochukwu et al. [15] carried out a study on the endemicity of schistosomiasis in some parts of Anambra State and reported 15.7% prevalence; similarly, Ekejindu et al. [16] reported urinary schistosomiasis prevalence of 11.8% in Anambra State. The low prevalence of schistosomiasis in Anambra is in contrast to what has been reported from some other parts of Nigeria. For example, Abdullahi et al. [17] reported 40.2% prevalence in Kano State, Nigeria. However, it is necessary to state that the focal epidemiology of schistosomiasis affects generalized conclusions on prevalence. Studies designed to focus on foci of the infections usually report higher prevalence than those designed otherwise. This study did not focus on the foci of infection, hence the need for a restrain in adopting the generalized 5.5% or 2.9% overall prevalence, especially because all the communities sampled were over one kilometer from water bodies.

The distribution of the infection based on occupation of the study participants, showed that fishers had the highest cases of the infection (57.1% and 71.4% from microscopy and dipstick, respectively). Fishing is conducted in water; this recurrent and probably frequent contact with water increases the chances of contact with the snail intermediate host of *S. haematobium* and the infective stages of the parasite. This is corroborated by the 103 greater odds of infection in fishers compared to students, which was also significant statistically (p < 0.05). Several studies have associated activities such as fishing, which required visit to water bodies recurrently and spending a long time inside the water [15,18]. From our study, visit to stream was not a sufficient risk for infection with urinary schistosomiasis. Implying that a mere visit to a stream may not be sufficient to cause infection. Performing activities in the water such as fishing and swimming were major factors associated with infection. “Bathe in stream” was similarly removed from the model due to redundancy in the presence of swimming in stream and fishing in stream. This may imply that “swimming in stream” instead of “bathe in stream” may better represent respondents’ bathing activities in water. The long-known importance of freshwater bodies to the epidemiology of schistosomiasis is the reason why control efforts globally have one of the core objectives as reducing contact with schistosome-infected water bodies. There is a need to design a procedure of schistosome control in Anambra State that will better address the concern of fishers. Telling them to quit fishing is not a good option.

Though prevalence of schistosomiasis in Anambra State is low, this occupationally related high prevalence of 57.1% to 71.4% among fishers, supported by the over 103 times greater odds of infection portends danger. The first source of concern is that there is the likelihood that prevalence of the infection at the foci of infection should be significantly higher than what our study has reported. Prevalence of *S. haematobium* infection is high where the people are in very close proximity to standing and infected freshwater and regularly come in contact with this water [16,19]. Prevalence of infection in such locality may approach a similar magnitude as reported for fishers in our study. In this study, sampling locations were not chosen with the intent to target areas where the appropriately conditions for spread of the disease (i.e., foci) existed. The second source of concern is that the low prevalence reported by this study and some other authors who adopted the same sampling procedure may present a statewide prevalence, which gives a tolerable level of the disease prevalence and unfortunately reduces the chances of access to treatment for the multitudes living in localities where very high prevalence occur. Therefore, a more humane approach based on the high prevalence among fishers suggests that future survey of prevalence should target the foci of infection.

The age group most affected by this infection from the study were those between ages 31–40 years and 41 years or older. There are no distinct reasons why these age groups had higher prevalence of infection. But from statistical analysis, we have reason to believe that fishers and farmers may fall more into these age categories. Inclusion of age along occupation degenerated our model, but inclusion of either played a similar role, though occupation was slightly better. More conservatively, maybe the prevalence of urinary schistosomiasis in the age groups 31–40 years and 41 years or older was skewed by the greater number of fishers and farmers in this age groups. The prevalence of the infection in males (2.9%) and females (2.9%) was identical. This suggests that there are no sex-biased tendencies to participate in activities that predispose to infection with *S. haematobium*. Males and females equally participate in activities such as bathing, swimming and washing in streams, which served as major predisposing factors to the infection. Sex-related behavioural differences and disparity in roles assigned males and females by some cultures are some factors that could cause sex-biased prevalence of urinary schistosomiasis in some populations [19]. Thus, these two factors could be said not to influence urinary schistosomiasis in the populations we studied. Similar conclusions have been drawn by other authors [17,18,19].

## Conclusion

It is evident from the results that the study area is endemic for urinary schistosomiasis. There is a need for focal studies, and there may probably be a need to design a health programs aimed at controlling the infection in focal areas in the state. The study also provides relevant information for designing a plan of action for the selective integrated and targeted control of urinary schistosomiasis in the local government areas. The GIS map produced contains information on the distribution of urinary schistosomiasis in Anambra State. Finally, the prevalence of *S. haematobium* infection together with the geographical location data of the area mapped can be used as an index for assessing the statewide prevalence of *S. haematobium* infection in Anambra State, Nigeria. The state government should ensure that pipe-borne water is provided for every community in the state. Drugs could also be provided for those suffering from the disease. Mass drug administration (MDA) targeted at the foci of infection may be needed. Where it is feasible, attention may be paid to targeting the intermediate hosts.

## Additional Files

The additional files for this article can be found as follows:

10.5334/aogh.2393.s1 Supplementary File 1. Figure S1.

10.5334/aogh.2393.s2 Supplementary File 2. Table S1.
