# Corrigendum: Mapping of Urinary Schistosomiasis in Anambra State, Nigeria

**DOI:** 10.5334/aogh.2540

**Published:** 2019-05-27

**Authors:** Yvonne E. Ndukwe, Rose N.N. Obiezue, Ifeanyi Oscar N. Aguzie, Joy T. Anunobi, Fabian C. Okafor

**Affiliations:** 1 Parasitology and Public Health Unit, Department of Zoology and Environmental Biology, University of Nigeria, Nsukka, Enugu State, NG; 2 Science Laboratory Technology Department, Federal Polytechnic, Idah, Kogi State, NG

## Abstract

This article details a correction to the article: Ndukwe, Y.E., Obiezue, R.N.N., Aguzie, I.O.N., Anunobi, J.T. and Okafor, F.C., 2019. Mapping of Urinary Schistosomiasis in Anambra State, Nigeria. *Annals of Global Health*, 85(1), p. 52. DOI: http://doi.org/10.5334/aogh.2393

## Corrigendum

It has come to the authors’ attention that there are three sections of Ndukwe et al. [1] that need to be corrected:

In the **statistical analysis section**, the sentence beginning ‘Mixed-effect logistic regression model…’ is corrected to:

*Binary logistic regression model was used to estimate the odds of infection with Schistosoma haematobium in relation to demographic and water contact activities*.

In the **statistical analysis section**, the sentence beginning ‘Univariate logistic regression was used…’ is corrected to:

*Bivariate logistic regression was used to estimate the crude odd ratios of infection associated with twelve variables (see Supplementary File 2)*.

In the **statistical analysis section**, the sentence beginning ‘“Age group” and “occupation” played similar roles…’ is corrected to:

*“Age group” and “occupation” played similar roles in the multivariate logistic regression model, which we understood to mean that the significant crude odds of infection in some age groups from our bivariate models may be due to occupation of people in those age groups*.
