# Supplementary figures and images for: Mapping of Urinary Schistosomiasis in Anambra State, Nigeria
Source: Ann Glob Health. 2019 Apr 2;85(1):52. doi: 10.5334/aogh.2393 (PMC6634351; doi:10.5334/aogh.2393)

Supplementary File 1 (S1)

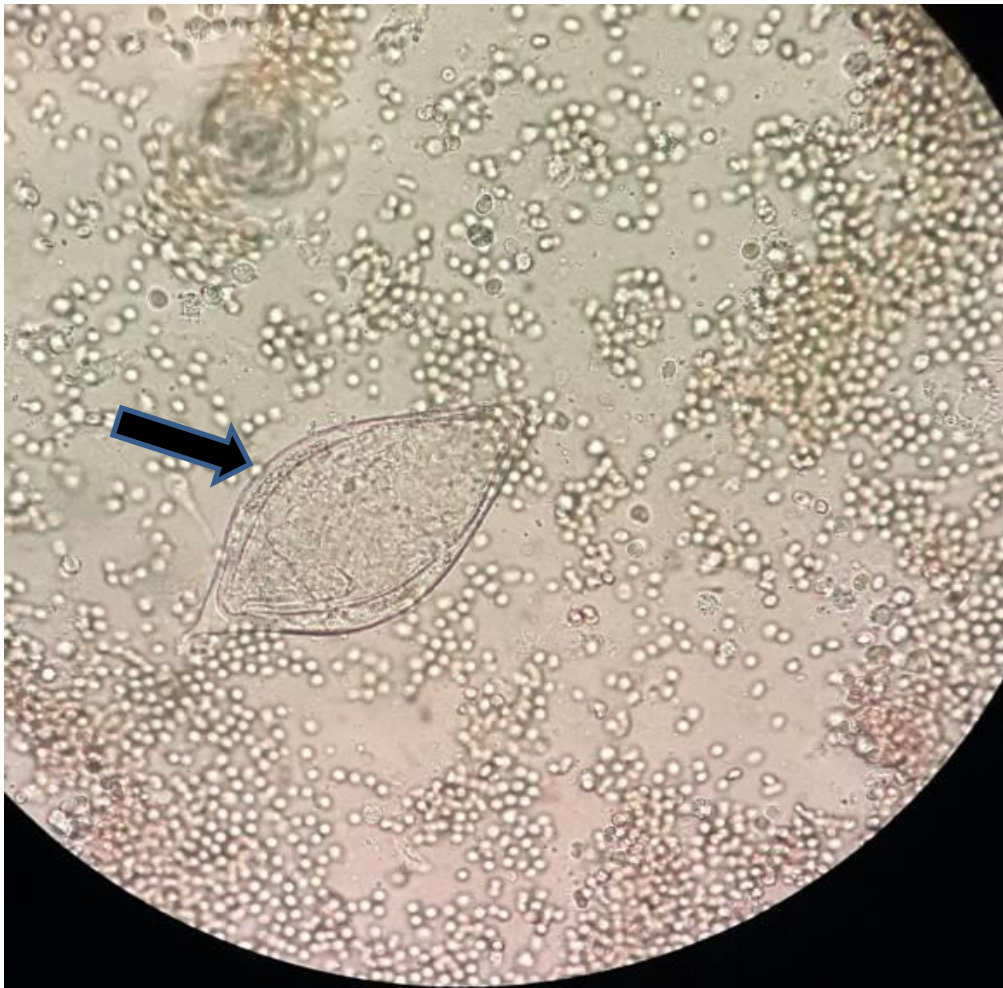

Figure S1: Eggs of *S. haematobium* as seen via the microscope.

Supplement: Supplementary File 1. — Figure S1. [file agh-85-1-2393-s1.pdf]
