# Supplementary material for: Mapping of Urinary Schistosomiasis in Anambra State, Nigeria
Source: Ann Glob Health. 2019 Apr 2;85(1):52. doi: 10.5334/aogh.2393 (PMC6634351; doi:10.5334/aogh.2393)
Supplement: Supplementary File 2. — Table S1. [file agh-85-1-2393-s2.pdf]

Supplementary File 2

Table S1: Crude odd ratios of variables in relation to urinary schistosomiasis infection in Anambra State based on microscopic detection of *Schistosoma haematobium* eggs in urine

**Variables**		**OR (95% CI)**	**P**
Senatorial District	Anambra Central	Reference	
	Anambra South	1.8621 (0.2153–15.4910)	0.551
	Anambra North	3.5660 (0.8992–23.6626)	0.108
Towns	Agulu	Reference	
	Ogidi	*	0.995
	Awka	*	0.995
	Ukpor	1.8750 (0.2141–16.4352)	0.542
	Ihiala	*	0.996
	Orafite	*	0.996
	Aguleri	1.3235 (0.2103–10.3874)	0.764
	Umueze-Anam	2.0089 (0.4117–14.4874)	0.417
	Omor	0.4327 (0.0197–4.6607)	0.500
Sex	Female	Reference	
	Male	1.4978 (0.4881–4.76607)	0.476
Age Groups (year)	0–10	Reference	
	11–20	2.6667 (0.5762–13.7781)	0.2053
	21–30	1.3636 (0.0666–10.9405)	0.7904
	31–40	10.5882 (1.8394–61.2326)	**0.0058**
	≥ 41	5.2174 (0.6614–33.1011)	0.0786
Occupation	Student	Reference	
	Civil servant	*	0.9905
	Farmer	4.6000 (0.6459–21.5064)	0.0722
	Trader	1.7037 (0.08849–10.8939)	0.6277
	Fishing	61.3333 (11.3377–378.0243)	< **0.00001**
Sources of drinking water	Tap water	Reference	
	Stream water	11.2000 (1.4822–59.6049)	**0.0070**
	Rain water	1.0769 (0.0567–5.9615)	0.9447
	Sachet water	*	0.9921

Bathe in stream	No	Reference	
	Yes	15.1042 (4.4757–68.7877)	< **0.00001**
Wash in stream	No	Reference	
	Yes	57.6393 (11.0512–105.9904)	**0.00013**
Fish in stream	No	Reference	
	Yes	19.8750 (6.1474–76.4082)	< **0.00001**
Swims in stream	No	Reference	
	Yes	24.9391 (4.8269–457.1084)	< **0.00001**
Visits stream	No	Reference	
	Yes	*	
Distance to stream	Far	Reference	
	Near	9.1299 (2.3148–47.0178)	**0.00305**

OR: odd ratio. CI: confidence interval. *odd ratio unavailable due to no infection in category; therefore odds not defined.
